# Roxadustat prevents Ang II hypertension by targeting angiotensin receptors and eNOS

**DOI:** 10.1172/jci.insight.133690

**Published:** 2021-09-22

**Authors:** Jing Yu, Shuqin Wang, Wei Shi, Wei Zhou, Yujia Niu, Songming Huang, Yue Zhang, Aihua Zhang, Zhanjun Jia

**Affiliations:** 1 Nanjing Key Laboratory of Pediatrics and; 2 Department of Nephrology, Children’s Hospital of Nanjing Medical University, Nanjing, China.; 3 Jiangsu Key Laboratory of Pediatrics, Nanjing Medical University, Nanjing, China.

**Keywords:** Nephrology, Vascular Biology, Drug therapy, Hypertension, Nitric oxide

## Abstract

The prevalence of hypertension is increasing globally, while strategies for prevention and treatment of hypertension remain limited. FG-4592 (Roxadustat) is a potentially novel, orally active small-molecule hypoxia-inducible factor (HIF) stabilizer and is being used clinically to treat chronic kidney disease (CKD) anemia. In the present study, we evaluate the effects of FG-4592 on hypertension. In an angiotensin II (Ang II) hypertension model, FG-4592 abolished hypertensive responses; prevented vascular thickening, cardiac hypertrophy, and kidney injury; downregulated AGTR1 expression; and enhanced AGTR2, endothelial NO synthase (eNOS), and HIF1α protein levels in the aortas of mice. Additionally, the levels of thiobarbituric acid reactive substances (TBARs) in blood and urine were diminished by FG-4592 treatment. In vascular smooth muscle cells, FG-4592 treatment reduced angiotensin receptor type 1 (AGTR1) and increased AGTR2 levels, while preventing Ang II–induced oxidative stress. In vascular endothelial cells, FG-4592 upregulated total and phosphorylated eNOS. Moreover, FG-4592 treatment was hypotensive in L-NAME–induced hypertension. In summary, FG-4592 treatment remarkably ameliorated hypertension and organ injury, possibly through stabilizing HIF1α and subsequently targeting eNOS, AGTR1, AGTR2, and oxidative stress. Therefore, in addition to its role in treating CKD anemia, FG-4592 could be explored as a treatment for hypertension associated with high renin angiotensin system (RAS) activity or eNOS defects.

## Graphical abstract

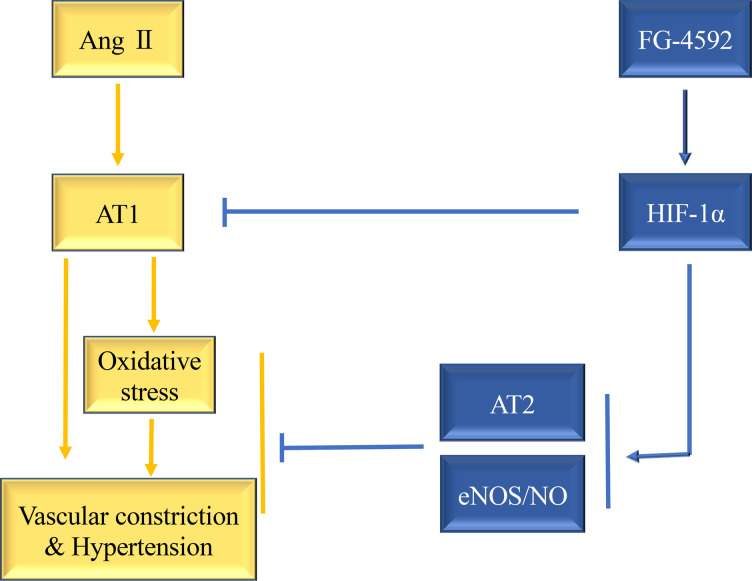

## Introduction

Hypertension is highly prevalent worldwide and contributes to the pathology of a number of clinical complications, including cardiovascular diseases and organ injuries, leading to increases in morbidity and mortality (1, 2). Mild high blood pressure generally does not cause obvious symptoms, while long-term high blood pressure is a major risk factor for cardiovascular disease, heart failure, chronic kidney disease (CKD), stroke, peripheral vascular disease, and dementia (3, 4). Currently, typical antihypertensive treatments include renin angiotensin system (RAS) inhibitors, calcium channel blockers, diuretics, β blockers, and α-receptor blockers. However, while there are multiple antihypertensive drugs available as treatment options in the clinic, hypertension control remains unsatisfactory, and improved treatments with both hypotensive and organ protective effects need to be developed.

Hypoxia-inducible factors (HIFs) are transcription factors that modulate the adaptation to hypoxia by driving the expression of genes contributing to glycolysis, hematopoiesis, angiogenesis, and vasculogenesis (5–8). HIFs operate as heterodimers composed of HIFα and HIFβ subunits that dimerize and bind to DNA. The HIF family includes 3 members: HIF1, HIF2, and HIF3. Conditional deletion of HIF1α from vascular smooth muscle cells (VSMCs) enhanced systolic, diastolic, and mean arterial pressure (MAP) in mice (9). Overexpression of HIF2α exacerbates pulmonary hypertension (10, 11). These reports indicate that HIF family members may be involved in blood pressure regulation. However, the activity of HIFs rapidly reduces under normoxic conditions, mediated by HIF prolyl hydroxylase domain proteins (HIF PHDs). HIF PHDs hydroxylate the α subunit of the HIF heterodimer at Pro-402 or Pro-564 within the C-terminal oxygen-dependent degradation domain. This hydroxylation causes the subunit to be a substrate of the von Hippel Lindau protein (VHL), an E3 ubiquitin ligase. Once poly-ubiquitinated by VHL, HIFα is transported to the proteasome for degradation, which ultimately inhibits the transcription of HIF-regulated genes (12, 13).

FG-4592, also known as Roxadustat, is a potentially novel, transient small-molecule inhibitor of HIF PHDs. FG-4592 is approved for the treatment of anemia in patients with CKD. FG-4592 treatment results in a transient burst of HIFα activity by inhibiting HIF PHDs and increasing endogenous erythropoietin (EPO) levels, thereby allowing erythropoiesis to occur (14–16). Since HIFs are known to regulate vascular tone, it is possible that FG-4592 may regulate blood pressure through its activity in increasing HIF activity. In this present study, we demonstrate that systemic administration of the HIFα stabilizer FG-4592 relieved hypertension and organ injury induced by angiotensin II (Ang II). Furthermore, our data suggest that the antihypertensive effects of FG-4592 may be mediated by enhancing endothelial NO synthase (eNOS) and differentially regulating angiotensin receptor type 1 (AGTR1) and AGTR2 in vasculature.

## Results

### FG-4592 treatment prevents Ang II–induced hypertension.

**Figure 1:**
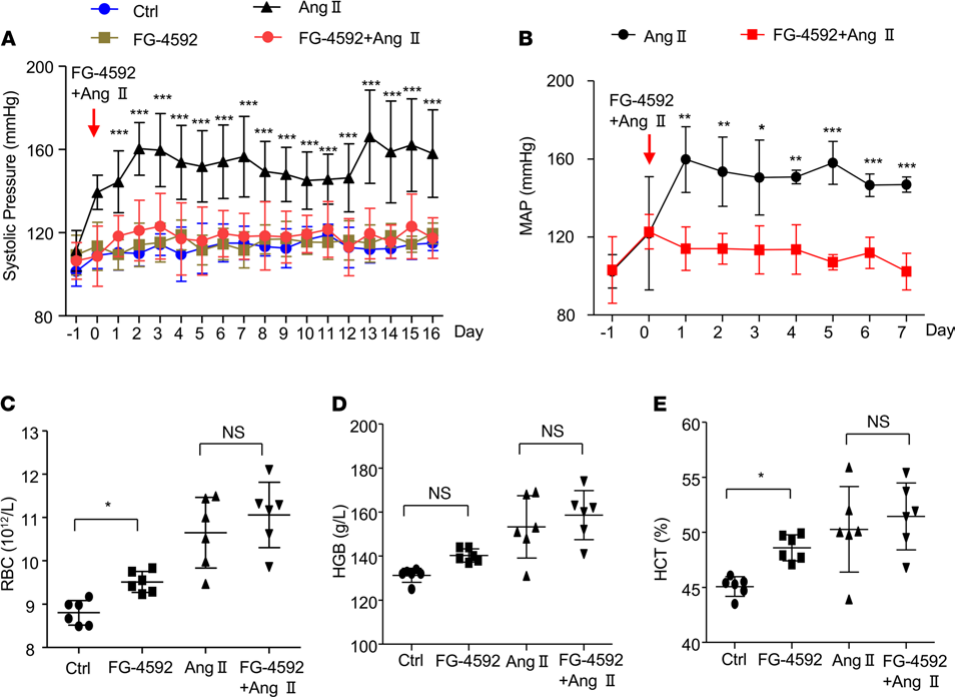
FG-4592 prevents Ang II–induced hypertension. (**A**) Systolic blood pressure measured by tail cuff for 16 days. Data are presented as mean ± SD (*n* = 8–10 per group). The mice treated with FG-4592 and Ang II showed a significant decrease in systolic pressure relative to the mice treated with Ang II alone. (**B**) MAP was measured by telemetry for 7 days. Data are presented as mean ± SD (*n* = 4 per group). The mice treated with FG-4592 and Ang II showed a significant decrease in MAP relative to the mice treated with Ang II alone. Significance values were determined by t test. (**C**–**E**) RGB (**C**), HG (**D**) and HCT (**E**) were detected in blood collected from mice at the end point of experiment. Data are presented as mean ± SD (*n* = 8–10 per group). In **A** and **C**–**E**, significance values were determined by 1-way ANOVA. Newman-Keuls multiple-comparison test was used for multiple comparisons. Ctrl = DMSO in saline. * *P* < 0.05, **P < 0.01, ***P < 0.001.

The effect of FG-4592 treatment on blood pressure was examined using a tail-cuff method in mice with hypertension induced by Ang II infusion. Mice treated with Ang II displayed higher systolic blood pressure, around 40 mmHg during the 16 days of administration. Treatment with FG-4592 almost completely abolished the hypertensive response of Ang II during the 16 days of blood pressure monitoring (Figure 1A). To confirm this phenomenon, telemetry was used to measure blood pressure. FG-4592 treatment also strikingly attenuated the prohypertensive activity of Ang II (Figure 1B). This was consistent with the blood pressure results determined by the tail-cuff method. FG-4592 is a drug used for treating CKD anemia, and the effects of FG-4592 on erythropoiesis were observed in mice with or without Ang II treatment. RBCs, hemoglobin (HG), and hematocrit (HCT) were mildly increased after FG-4592 treatment with or without Ang II infusion (Figure 1, C–E).

### FG-4592 treatment attenuates Ang II–induced hypertrophic effects on mouse aorta and heart tissue.

**Figure 2:**
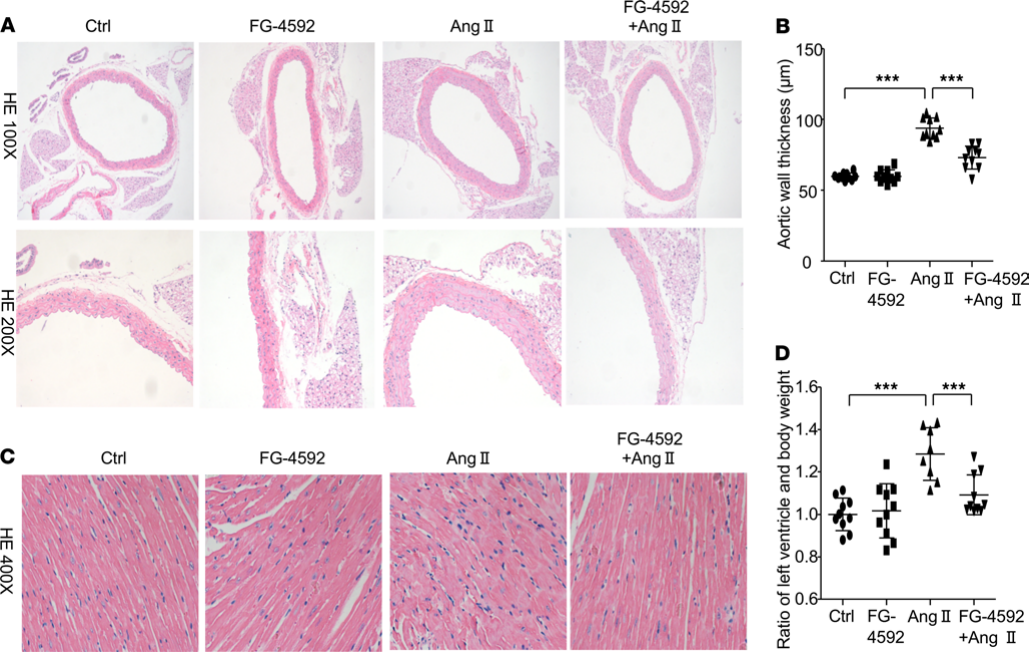
FG-4592 prevents Ang II–induced hypertrophic effects on aorta and heart tissue in mice. (**A**) Representative images of H&E staining (total original magnification: [upper] ×100, [lower] ×200) of aorta tissue. (**B**) Quantification of aortic wall thickness in each group. (**C**) Representative images of H&E staining of heart tissue in each group (total original magnification, ×200). (**D**) Quantification of left ventricle to body weight ratio. Data are presented as mean ± SD (*n* = 8–10 per group). Significance values were determined by 1-way ANOVA. Newman-Keuls multiple-comparison test was used for multiple comparisons. Ctrl, DMSO in saline. ***P < 0.001.

Consistent with the blood pressure data, histological analysis of aorta tissues by H&E staining exhibited marked attenuation of aortic wall thickening in Ang II–treated mice following FG-4592 administration (Figure 2A). Mice treated with Ang II displayed a 57% increase in aortic wall thickness compared with WT mice, which was diminished by FG-4592 treatment (Figure 2B). In addition to aortic wall remodeling, both pressure overload and Ang II infusion result in cardiac hypertrophy. Thus, we further evaluated the hypertrophic effect of Ang II infusion on murine hearts and found that Ang II infusion–induced cardiomyocyte hypertrophy (indicated by H&E staining) was substantially relieved by the administration of FG-4592 (Figure 2C). Meanwhile, mice treated with Ang II displayed a 28% increased ratio of left ventricle to body weight; this was ameliorated by treatment with FG-4592 (Figure 2D). These data from aorta and heart tissue demonstrate that FG-4592 treatment prevents remodeling of vascular and cardiac tissue induced by Ang II, possibly by lowering blood pressure and attenuating cellular responses.

### FG-4592 treatment ameliorates Ang II–induced glomerular injury in mice.

**Figure 3:**
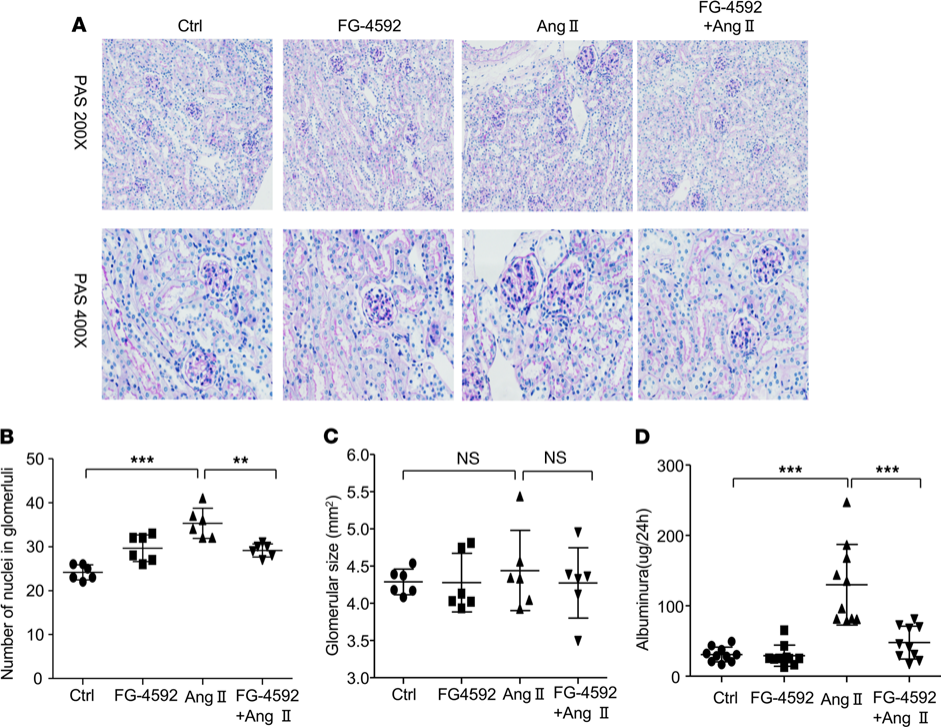
FG-4592 attenuates Ang II–induced glomerular injury in mice. (**A**) Representative images of PAS staining of kidneys in each group (total original magnification, [upper] ×200, [lower] ×400). (**B**) Quantification of the number of nuclei in glomeruli. Data are presented as mean ± SD (*n* = 6 per group). (**C**) Quantification of glomerular size. Data are presented as mean ± SD (*n* = 6 per group). (**D**) Quantification of albuminuria. Data are presented as mean ± SD (*n* = 10 per group). Significance values were determined by 1-way ANOVA. Newman-Keuls multiple-comparison test was used for multiple comparisons. Ctr, DMSO in saline. **P < 0.01, ***P < 0.001.

We evaluated the effects of FG-4592 on renal injury in Ang II–treated mice. Mesangial cell proliferation, glomerular size, and proteinuria were evaluated as markers of renal injury. Histologically, mice treated with Ang II displayed mesangial cell proliferation, which was alleviated after treatment with FG-4592 (Figure 3, A and B). No significant effects of FG-4592 treatment were observed on glomerular size with or without Ang II treatment (Figure 3C). In concordance with the amelioration of glomerular mesangial cell proliferation, FG-4592 treatment largely abolished proteinuria in Ang II–treated mice (Figure 3D). These data suggest that FG-4592 induces a renal protective effect by antagonizing Ang II hypertension–associated renal injury.

### Effects of FG-4592 treatment on urine volume and urinary electrolyte output in Ang II–treated mice.

**Figure 4:**
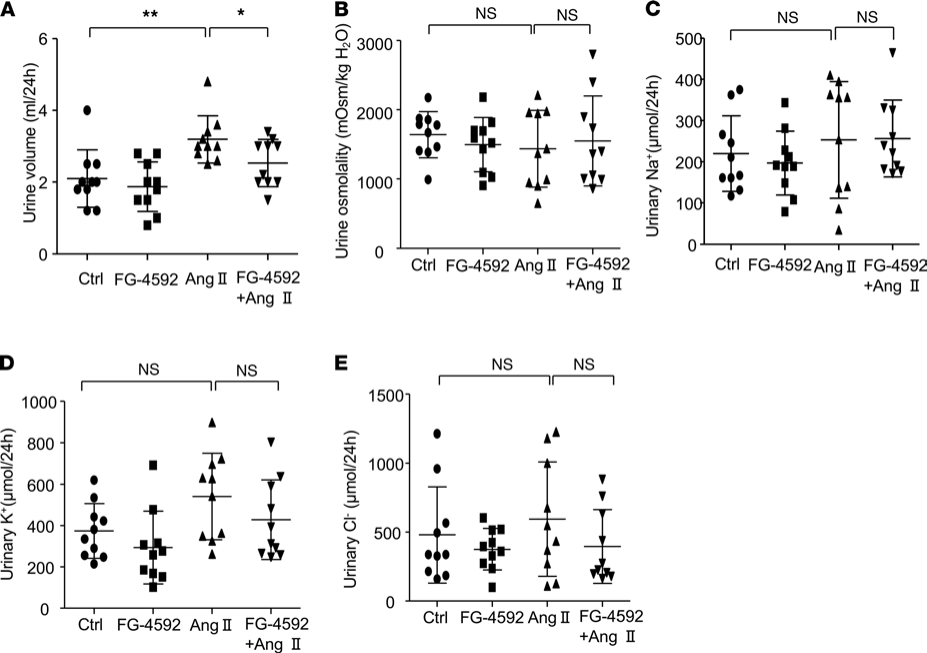
Effects of FG-4592 on urine volume and urinary electrolyte output in Ang II–treated mice. (**A**) Urine volume. (**B**) Urinary osmolality. (**C**) Urinary Na^+^ excretion. (**D**) Urinary K^+^ excretion. (**E**) Urinary Cl^–^ excretion. Data are presented as mean ± SD (*n* = 10 per group). Significance values were determined by 1-way ANOVA. Newman-Keuls multiple-comparison test was used for multiple comparisons. Ctrl , DMSO in saline. *P < 0.05, **P < 0.01.

In addition to vascular tone, blood pressure can be regulated by blood volume, which is highly associated with water and salt balance. Therefore, we evaluated the effects of FG-4592 treatment on urine volume and electrolyte output. Urine was collected from mice at the end point of the experiment, and urine osmolality and urinary electrolyte levels were measured and calculated. No significant effects of FG-4592 treatment were observed on urine volume, urine osmolality, and urinary electrolyte output (Na^+^, K^+^, and Cl^–^) in mice with or without Ang II infusion (Figure 4, A–E), suggesting that the antihypertensive role of FG-4592 in this hypertension model could be independent of the regulation of water and salt metabolism.

### FG-4592 treatment attenuates Ang II–induced oxidative stress in mice.

**Figure 5:**
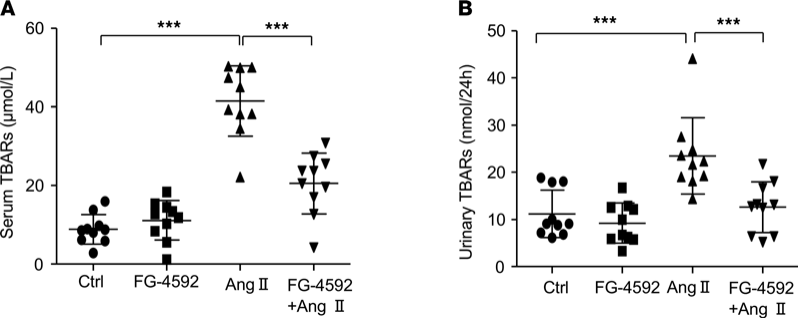
Oxidative stress induced by Ang II is blocked by FG-4592 administration in mice. (**A**) Serum levels of TBARs (μmol/L). (**B**) Quantification of urinary TBARs (nmol/24 hours). Data are presented as mean ± SD (*n* = 10 per group). Significance values were determined by 1-way ANOVA. Newman-Keuls multiple-comparison test was used for multiple comparisons. Ctrl, DMSO in saline. ***P < 0.001.

Ang II hypertension is highly associated with oxidative stress. In order to determine the effects of Ang II infusion and FG-4592 treatment on oxidative stress, we measured thiobarbituric acid reactive substance (TBAR) levels in blood and urine. Ang II treatment led to a 72% increase of TBARs in blood, which was almost completely abolished by FG-4592 treatment (Figure 5A). A similar result was observed in urine (Figure 5B). These findings indicated that FG-4592 has strong antioxidative properties, which could contribute to the blood pressure–lowering effect of this antianemia drug.

### Effects of FG-4592 treatment on the regulation of HIF1α, AGTR1, AGTR2, eNOS, and p-eNOS in aorta tissue of mice challenged with Ang II.

**Figure 6:**
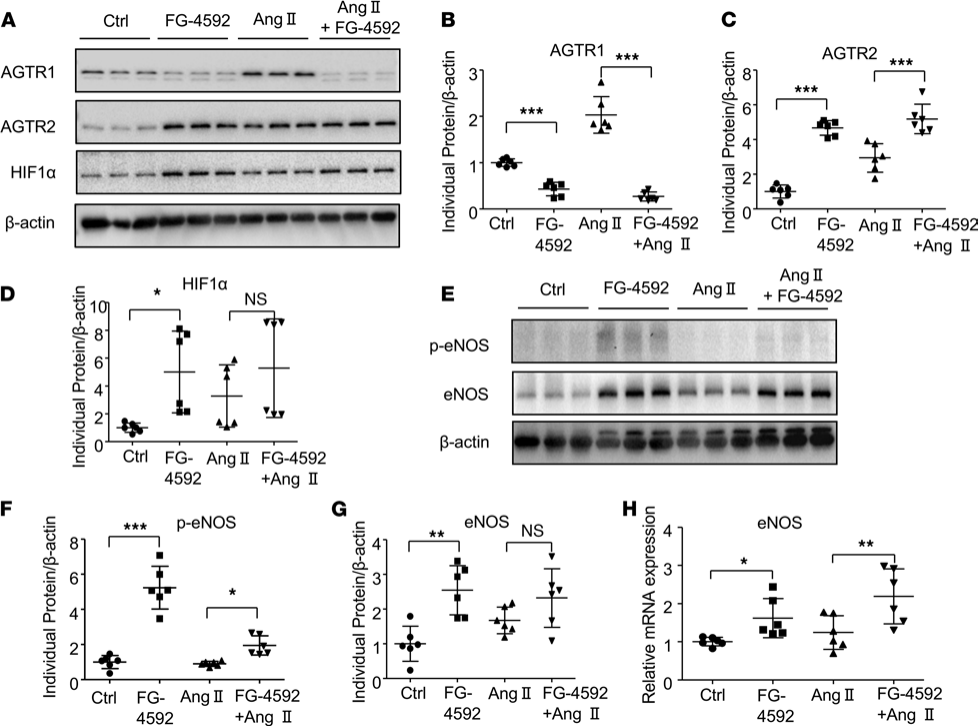
Effects of FG-4592 on the regulation of AGTR1, AGTR2, eNOS, p-eNOS, and HIF1α in aorta tissue of mice challenged with Ang II. (**A**). Representative expression of AGTR1, AGTR2, and HIF1α in aorta tissue. (**B**–**D**) Quantification of AGTR1 (**B**), AGTR2 (**C**), and HIF1α (**D**) in mouse aorta tissue. (**E**) Representative expression of p-eNOS and eNOS in aorta tissue. (**F** and **G**) Quantification of p-eNOS (**F**) and eNOS (**G**) in aorta tissue of mice. (**H**) The mRNA expression of eNOS was detected in aorta tissue. Data are presented as mean ± SD (*n* = 6 per group). Significance values were determined by 1-way ANOVA. Newman-Keuls multiple-comparison test was used for multiple comparisons. Ctrl, DMSO in saline. *P < 0.05, **P < 0.01, ***P < 0.001.

The regulation of blood pressure by Ang II is mediated by signaling through the Ang II receptors. Therefore, we evaluated the expression of AGTR1 and AGTR2 in aorta tissue after FG-4592 treatment. FG-4592 is a stabilizer of HIFα, and we observed an increase in the abundance of HIF1α in aorta tissue after FG-4592 treatment (Figure 6,A and D). Surprisingly, FG-4592 treatment alone lowered AGTR1 but enhanced AGTR2 expression (Figure 6, A–C). Similarly, FG-4592 also lowered AGTR1 and increased AGTR2 expression in aorta tissue of Ang II–treated mice (Figure 6, A–C). These data suggest that FG-4592 may rebalance Ang II receptors in the vasculature. NO is also involved in vasodilatation and blood pressure regulation, and the *eNOS* gene is a known transcriptional target of HIF1α. Therefore, we examined the expression of eNOS in aorta tissue and found that both eNOS mRNA and protein expression were upregulated by FG-4592 in mice with or without Ang II infusion (Figure 6, E and H). The protein expression of phosphorylated eNOS (p-eNOS) was also increased after FG-4592 administration (Figure 6, E–G). These data indicated that FG-4592 can enhance HIF1α expression to regulate Ang II receptors and eNOS to prevent hypertension induced by Ang II.

### Effects of FG-4592 on the regulation of AGTR1, AGTR2, eNOS, p-eNOS, and HIF1α in VSMCs and endothelial cells.

**Figure 7:**
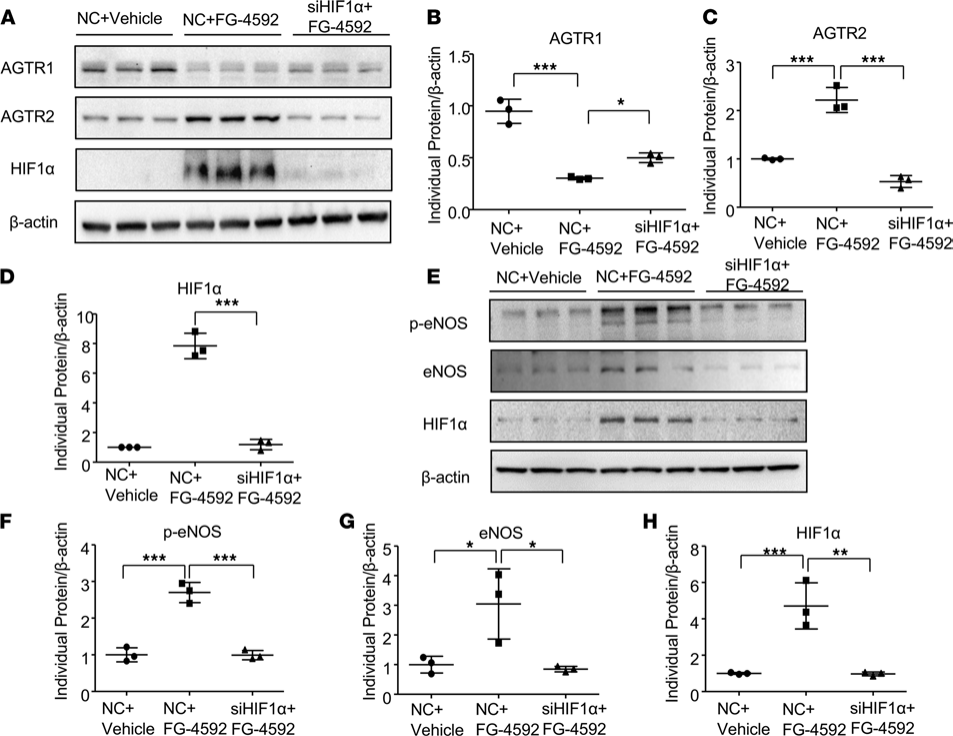
Effects of FG-4592 on the regulation of AGTR1, AGTR2, eNOS, p-eNOS, and HIF1α in cultured cells. (**A**) Smooth muscle cells were treated with FG-4592 and HIF1α siRNA. AGTR1, AGTR2, and HIF1α expressions were evaluated by immunoblotting. (**B**–**D**) Quantification of immunoblotting analysis from **A**. (**E**) Endothelial cells were treated with FG-4592 and HIF1α siRNA. p-eNOS, eNOS, and HIF1α expressions were evaluated by immunoblotting. (**F**–**H**) Quantification of immunoblotting analysis from (**E**). β-Actin was used as loading control. Data are presented as mean ± SD (*n* = 3 per group). Significance values were determined by 1-way ANOVA. Newman-Keuls multiple-comparison test was used for multiple comparisons. NC, negative control (a control of scrambled siRNA). Vehicle, DMSO in DMEM medium. *P < 0.05, **P < 0.01, ***P < 0.001.

We further evaluated the effects of FG-4592 on the regulation of Ang II receptors in cultured SMCs. FG-4592 treatment not only increased the abundance of HIF1α, but also led to significant downregulation of AGTR1 and increased AGTR2 expression (Figure 7, A–D). Next, we used an siRNA approach to downregulate HIF1α to evaluate the potential role of HIF1α in the regulation of AGTR1 and AGTR2. Downregulation of HIF1α in SMCs increased AGTR1 and decreased AGTR2 (Figure 7, A–D). In endothelial cells, FG-4592 enhanced the expression of eNOS, p-eNOS, and HIF1α; this expression was entirely abolished by silencing HIF1α (Figure 7, E–H). These data provide evidence that FG-4592 regulates Ang II receptors and eNOS through HIF1α.

### HIF1α and PPARγ regulate AGTR1 and AGTR2 expression.

**Figure 8:**
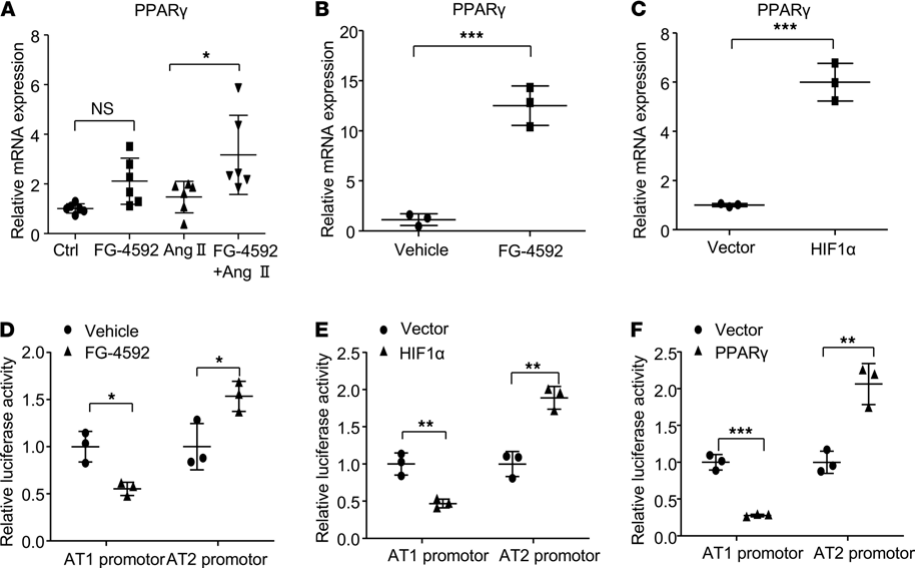
Expression of AT1 and AT2 are regulated by HIF1α and PPARγ. (**A**) The mRNA expression of PPARγ in the aorta tissue was evaluated. Data are presented as mean ± SD (*n* = 6 per group). Significance values were determined by 1-way ANOVA. Newman-Keuls multiple-comparison test was used for multiple comparisons. Ctrl, DMSO in saline. (**B**) Smooth muscle cells were treated with FG-4592, and the mRNA expression of PPARγ was evaluated by qPCR. (**C**) Smooth muscle cells were transfected with HIF1α expression plasmids, and the mRNA expression of PPARγ was evaluated by qPCR. (**D** and **E**) Promotor activity from the AGTR1 and AGTR2 promoters were evaluated by dual-luciferase assays after treatment with FG-4592 (**D**) or transfection of HIF1α (**E**) or transfection of PPARγ (**F**). (**B**–**F**) Data are presented as mean ± SD (*n* = 3 per group). Significance values were determined by *t* test. Vector, empty vector. Vehicle, DMSO in DMEM medium. *P < 0.05, **P < 0.01, ***P < 0.001.

PPARγ also plays an important role in regulating vascular tone, and we examined the effects of FG-4592 on regulating PPARγ expression. FG-4592 significantly enhanced the mRNA expression of PPARγ in aorta tissue of mice treated with or without Ang II (Figure 8A). Likewise, FG-4592 treatment increased PPARγ expression in cultured SMCs. The mRNA expression of PPARγ was upregulated by FG-4592 treatment or by overexpression of HIF1α (Figure 8, B and C). To further determine whether HIF1α and PPARγ regulate AGTR1 and AGTR2 expression at the transcriptional level, we used a dual luciferase assay. FG-4592 treatment alone led to a significant reduction of luciferase activity from the AGTR1 promotor, and it enhanced luciferase activity from the AGTR2 promotor (Figure 8D). The same results were observed by overexpression of HIF1α or PPARγ (Figure 8, E and F). These data suggest that both HIF1α and PPARγ regulate AGTR1 and AGTR2 expression at the transcriptional level, possibly via a positive link between HIF1α and PPARγ.

### FG-4592 attenuates Ang II–induced oxidative stress in VSMCs.

**Figure 9:**
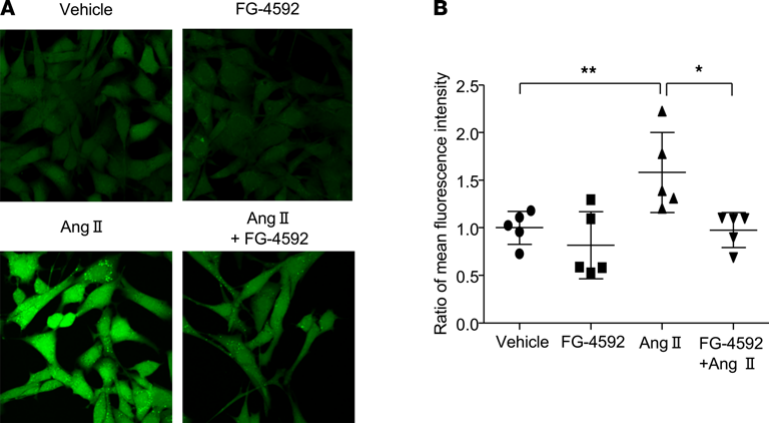
Oxidative stress induced by Ang II is blocked by FG-4592 administration in cultured smooth muscle cells. (**A**) Representative immunofluorescence staining for ROS production in cultured smooth muscle cells. (**B**) FACS analysis of ROS production in cultured smooth muscle cells. Data are presented as mean ± SD (*n* = 5 per group). Significance values were determined by 1-way ANOVA. Newman-Keuls multiple-comparison test was used for multiple comparisons. Vehicle, DMSO in DMEM medium. *P < 0.05, **P < 0.01. Total original magnification, x400.

Because we observed an antioxidative effect of FG-4592 in vivo, we explored the antioxidative effect of FG-4592 on cultured SMCs. By immunofluorescence staining and FACS, we observed that Ang II–induced oxidative stress in VSMCs was significantly blocked by FG-4592 treatment in vitro (Figure 9, A and B).

### FG-4592 attenuates L-NAME–induced hypertension in mice.

To further identify the role of eNOS/NO in blood pressure regulation by FG-4592, L-NAME — a known inhibitor of NO production — was used to induce hypertension in mice. Mice treated with L-NAME displayed a 20% increase in blood pressure, and treatment with FG-4592 resulted in a significant decrease in blood pressure (Supplemental Figure 1A; supplemental material available online with this article; https://doi.org/10.1172/jci.insight.133690DS1). eNOS can be transcriptionally regulated by HIF1α, which is stabilized by FG-4592, and we therefore evaluated the mRNA expression of eNOS. We found that FG-4592 treatment significantly upregulated the mRNA expression of eNOS (Supplemental Figure 1B). In line with the hypotensive effects of FG-4592, reduced urinary NO production in L-NAME–treated mice was restored by administration of FG-4592 (Supplemental Figure 1C). Similarly, reduced urine volume in L-NAME–treated mice was also reversed after FG-4592 treatment (Supplemental Figure 1D). This partial effect of FG-4592 in lowering L-NAME–induced high blood pressure not only suggested a hypotensive role of FG-4592 in a different hypertension model, but also indicated that eNOS might contribute to the antihypertensive effects of FG-4592 on Ang II hypertension.

### Safety evaluation of FG-4592 on organ functions in mice with or without Ang II treatment.

We also evaluated the effects of FG-4592 treatment on murine organ functions by measuring liver enzyme levels, blood urea nitrogen (BUN), and serum creatinine (Cr). We found that alanine aminotransferase (ALT), aspartate aminotransferase (AST), lactate dehydrogenase (LDH), BUN, and Cr were not affected by FG-4592 treatment in mice with or without Ang II treatment (Supplemental Figure 2, A–E). These data largely suggest that FG-4592 could be well tolerated at the current dose and treatment protocol we used.

## Discussion

Hypertension is a common clinical complication, and HIFs are involved in regulating blood pressure (7, 9, 17, 18). FG-4592 is a potentially novel, orally active small-molecule inhibitor of HIF PHD. FG-4592 is currently used for the clinical treatment of CKD anemia by promoting endogenous EPO synthesis (14, 16). In the present study, we evaluated the effects of FG-4592 on Ang II–induced hypertension. Our major finding was that FG-4592 treatment largely abolished Ang II–induced hypertension and markedly alleviated organ injury and oxidative stress.

The pathogenesis of human hypertension remains elusive, as clear genetic or mechanistic causes of hypertension have not yet been clearly defined. The past decade has not seen significant development of new therapeutic drugs to treat hypertension, and the application of old hypotensive drugs does not adequately control blood pressure nor protect organs for many patients. Therefore, it is our goal to find new candidate drugs to treat hypertension. In our present study, FG-4592 alleviated Ang II–induced hypertension and protected against organ injury, suggesting the potential for FG-4592 as a treatment for hypertension associated with high RAS activity.

Ang II transduces signaling and downstream functions by directly binding to and activating 2 GPCRs, AGTR1 and AGTR2 (19, 20). These 2 receptors oppose each other in function. The functions of Ang II — including vasoconstriction, cellular dedifferentiation and growth, and salt and water reabsorption that lead to hypertension — are mediated through AGTR1 (21–23). On the other hand, activation of AGTR2 induces cellular differentiation, growth inhibition, and vasodilation (19, 24). Because the expression of AGTR1 is much more abundant than AGTR2 in cardiovascular and renal tissues, the action of AGTR1 generally predominates in vivo. However, the effects of AGTR2 activation can be observed in vivo when RAS is activated or when AGTR1 activation is blocked (25, 26).

In this study, FG-4592 demonstrated potent antihypertensive property in Ang II–infused mice. Interestingly, we observed that AGTR1 expression was decreased in aorta tissue after FG-4592 administration, but AGTR2 was upregulated in aorta tissue following FG-4592 treatment. Furthermore, treatment of VSMCs with FG-4592 in vitro resulted in the same phenomenon of AGTR1/AGTR2 regulation as the aortic tissue in vivo. These results support a role of FG-4592 in modulating the balance of AGTR1 and AGTR2 expression in vascular tissue.

FG-4592 is a stabilizer of HIFα family proteins. The HIFα family contains 3 members: HIF1α, HIF2α, and HIF3α. The biological roles of HIF1α and HIF2α vary depending upon the microenvironment and tissue context (27–30). SMC-HIF1α–KO mice display the specific loss of HIF1α from VSMCs, and they exhibit vascular contraction and increased blood pressure in response to Ang II administration, along with increased vascular AGTR1 expression (9). Although pulmonary HTN was reported in endothelial-HIF2α–KO mice (10, 31), systemic high blood pressure in endothelial-HIF2α–KO mice was not observed (9). The role of HIF3α in regulation of blood pressure has not yet been reported. In the present study, silencing HIF1α blocked FG-4592–mediated upregulation of AGTR2, eNOS, and p-eNOS, as well as FG-4592–mediated downregulation of AGTR1. Thus, based on previous reports and our own data, enhancement of HIF1α by FG-4592 treatment could play a role in regulating blood pressure, possibly by regulating the expression of AGTRs, as well as eNOS.

HIF not only mediates acute/immediate responses to changes in oxygen tension, but it also controls the expression of genes regulating vasodilation and vasoconstriction (32–34). Furthermore, substances released by endothelial cells play important roles in regulating vascular tone. Endothelium-derived NO is a potent biological mediator of vasodilatation (35, 36). As a transcriptional target gene of HIF1α, eNOS is increased in aorta tissue, and p-eNOS is enhanced after FG-4592 treatment in mice, indicating that HIF1α-regulated eNOS/p-eNOS may mediate FG-4592 effect against Ang II–induced hypertension.

We further explored the role of FG-4592 in lowering blood pressure in an L-NAME model of hypertension. In this model, we also evaluated the potential contribution of eNOS/NO to the blood pressure-lowering effect of FG-4592. In the L-NAME model, FG-4592 treatment only partially lowered blood pressure. This partial effect on lowering L-NAME–induced hypertension was accompanied with the partial restoration of reduced NO in urine, indicating that FG-4592 at the current dose could significantly, but not entirely, prevent hypertensive effects induced by the eNOS inhibitor L-NAME. These data demonstrate that the relationship between AGTRs, eNOS, and oxidative stress need further evaluation.

Two recent clinical trials published in the New England Journal of Medicine did not report a hypotensive effect of FG-4592 in patients with end-stage renal disease, either with or without dialysis (14). These clinical results might be explained by the routine clinical administration of angiotensin-converting enzyme inhibitor (ACEI) or angiotensin receptor blocker (ARB) drugs in patients with end-stage renal disease. FG-4592 may also mainly target RAS, as RAS inhibitors lead to additive effects on lowering blood pressure in these patients. Moreover, the intermittent administration of FG-4592 for the treatment of CKD anemia might not provide persistent regulation of the signaling pathways regulating blood pressure. In the future, a clinical trial of FG-4592 in individuals with hypertension is warranted to clarify the role of this drug in treating hypertension in human patients.

In summary, the present study demonstrated a potent effect of FG-4592 against Ang II–induced hypertension. The antihypertensive effects of FG-4592 may be mediated through upregulation of AGTR2 and eNOS, downregulation of AGTR1, and inhibition of oxidative stress. Due to the approved clinical use of FG-4592, our findings suggest the clinical potential of this antianemia drug in treating hypertension associated with RAS activation or eNOS defects.

## Methods

### Antibodies and kits.

Antibodies against AGTR1 (catalog ab124734, 1:1000), AGTR2 (catalog ab92445, 1:1000), eNOS (catalog ab199956, 1:1000), p-eNOS (catalog ab215717, 1:1000), and HIF1α (catalog ab179483, 1:1000) were bought from Abcam. Antibodies against β-actin (catalog ap0060, 1:2000) were purchased from Bioworld. Reactive oxygen assay kits were purchased from Biyuntian. TBARs assay kits were purchased from Cayman. The nitrate oxide/nitrite (NOx) assay kits were purchased from Cayman. L-NAME was purchased from MCE. FG-4592 (Roxadustat), the HIF prolyl hydroxylase inhibitor, was purchased from Selleck Chemicals.

### Murine hypertension models.

For Ang II hypertension model, microcosmic pumps were implanted s.c. in the midscapular region of male adult (8 weeks old) C57BL/6 mice. The osmotic pumps delivered Ang II at a rate of 1.4 mg/kg per day. For the L-NAME–induced hypertension model, L-NAME was dissolved in drinking water and delivered to male adult (8 weeks old) C57BL/6 mice at a concentration of 1.3 mg/mL. FG-4592 was delivered at 10 mg/kg daily by i.p. injection. All mice were purchased from the model animal research center of Nanjing Medical University and were maintained on a 12-hour light/dark cycle in a temperature-controlled (19°C–21°C) room. Mice were fed standard rodent chow with ad libitum access to drinking water. At the experimental end point, urine was collected using metabolic cages. Then, the mice were anesthetized using isoflurane and blood samples were collected from the posterior vena cava using 25 G needles. After collecting the blood, the mice were euthanized by cervical dislocation, and tissues (kidney, heart, and aorta) were collected for further analysis.

### Blood pressure measurement.

Systolic blood pressure was measured by the tail-cuff method using a Visitech BP2000 Blood Pressure Analysis System (Apex). All animals were habituated to the blood pressure measurement device for 7 days. Animals underwent 1 cycle of 12 measurements reordered per day for 10 or 17 days. For the telemetry method of monitoring blood pressure, mice (8 weeks old) were anesthetized with isofluorane; then, a catheter was inserted into the ascending aorta through catheterization of the carotid artery to monitor MAP. After collection of baseline MAP, mice were implanted with an osmotic minipump driving infusion of vehicle or Ang II (37). The minipump was placed under the skin of the flank region of mice. Administration of FG-4592 or vehicle was started from the day that the minipump was inserted and was continued until the end of experiment.

### Cell culture.

Mouse endothelial cells and SMCs were obtained from the American Type Culture Collection (ATCC) and were cultured in DMEM medium supplemented with 10% heat-inactivated FBS (Thermo Fisher Scientific), 100 U/mL penicillin, and 100 μg/mL streptomycin at 37°C in the presence of 5% CO_2_ (Invitrogen).

### Histology.

Aorta, heart, and kidney tissues were fixed in 10% (vol/vol) formalin overnight and embedded into paraffin blocks. Transverse sections were cut and stained with H&E or periodic acid–Schiff (PAS).

### Immunoblotting.

Lysates of aortas and cultured cells were subjected to SDS-PAGE and electrophoretically transferred to PVDF membranes. The membranes were exposed to primary antibodies overnight at 4°C. After incubation with the appropriate peroxidase-linked secondary antibody for 1 hour at room temperature, immunoreactive proteins were visualized using a chemiluminescence reaction kit (Chemicon). The results were quantified by densitometry in the linear range of membrane exposure using Image lab software (Canon) and normalized to loading control protein expression.

### Quantitative PCR (qPCR).

Total RNA from cultured cells and ex vivo tissues were extracted using TRIzol reagent (Invitrogen). Primers were designed using Primer3 software (available at http://frodo.wi.mit.edu/) and synthesized by Huada Gene. The primer sequences used are shown as follows: eNOS, 5′-CGTCCTGCAAACCGTCAGA-3′ (sense) and 5′-TCCTGGGTG CGCAATGTGAG-3′ (antisense); PPARγ, 5′-CTTTATGGAGCCTAAGTTTGAGTT T-3′ (sense) and 5′-CAGCAGGTTGTCTTGGATGT-3′ (antisense); GAPDH, 5′-GTC TTCACTACCATGGAGAAGG-3′ (sense) and 5′-TCATGGATGACCTTGGCCAG -3′ (antisense). Reverse transcription was performed using Transcriptor First Stand cDNA Synthesis kit (Takara) according to the manufacturer’s instructions. qPCR amplification was performed using the ABI 7500 Real-Time PCR Detection System with Fast Start Universal SYBR green master mix (Vazyme). Cycling conditions were 95°C for 10 minutes, followed by 40 repeats of 95°C for 15 seconds and 60°C for 1 minute. The relative gene expression levels were calculated using the ΔΔCt method (where ΔCt is threshold cycle), and GAPDH was used as the internal control.

### Transfection of siRNA.

Scrambled siRNA and HIF1α siRNA were transfected into SMCs or endothelial cells seeded in 6-well plates using Lipofectamine 2000 (Invitrogen) according to the manufacturer’s instructions. The control of scrambled siRNA was used as a negative control (NC). After 24-hour siRNA transfection, FG-4592 was added to the cells, and DMSO in medium was used as a vehicle control. The cells were incubated with FG-4592 or vehicle for another 24 hours. Then, lysates of these cells were subjected to immunoblotting.

### Dual-luciferase reporter assay.

Firefly luciferase reporter plasmid containing the promotor region of human AGTR1 or AGTR2 was cotransfected with *Renilla* plasmid into 293T cells, which had been seeded in 24-well plates, using Lipofectamine 2000 (Invitrogen) according to the manufacturer’s instructions. After 24 hours, FG-4592 was added to the cells, and the cells were incubated for another 24 hours. 293T cell culture medium DMEM containing DMSO was used as a vehicle control. Finally, passive lysis buffer was added to lyse cells, and the lysate was collected. Firefly and *Renilla* luminescence signals were detected according to the manufacturer’s instructions (Promega). The promotor activity of AGTR1 and AGTR2, indicating by firefly luminescence signal, were normalized to the control *Renilla* luminescence signal. To detect the effects of HIF1α or PPARγ on the promoter activity of AGTR1 and AGTR2, the firefly luciferase reporter plasmid containing the promotor area of human AGTR1 or AGTR2 was cotransfected with HIF1α or PPARγ expression plasmids into 293T cells, as above. The firefly luciferase reporter plasmid containing the promotor area of human AGTR1 or AGTR2 cotransfected with an empty plasmid was used as a control. After 48 hours, passive lysis buffer was added to the cells, and the lysate was collected. Promoter activity was determined was as above.

### Flow cytometric analysis of ROS production.

The fluorogenic substrate 2’,7’-dichlorofluorescein diacetate (DCFDA) is a cell-permeable dye that is oxidized to the highly fluorescent 2’,7’- dichlorofluorescein (DCF) by reactive oxygen species (ROS) and can, therefore, be used to monitor levels of intracellular ROS. The DCFDA reagent was purchased from Biyuntian. For measurement of ROS, cells were grown on 12-well plates and subjected to treatment of Ang II or FG-4592. After 24 hours, cells were washed twice with DMEM and incubated for 30 minutes with 10 μM DCFDA and then treated with Ang II in the presence or absence of FG-4592. At the end of the incubation period, the cells were washed twice with DMEM and collected using 0.25% trypsin. Stained cells were analyzed using a BD FACS Caliber flow cytometer, and data analysis was performed with FlowJo software.

### Measurements of urinary nitrate/nitrite.

A nitrate oxide/nitrite (NOx) assay kit was purchased from Cayman and was used according to the manufacturer’s instructions. Briefly, 80 μL of centrifuged (12,000*g* for 5 minutes at 4°C) urine samples was added to the wells of a 96-well plate, followed by addition of 10 μL of each of the Enzyme Cofactor mixture and the Nitrate Reductase mixture, containing lyophilized NAPDH to reconstitute the cofactor by addition of 1.2 mL of assay buffer (20 mM potassium phosphate, PH 7.4). The plate was covered and incubated at room temperature for 1 hour, followed by sequential addition of 50 μL of Griess Reagent R1 and the same amount of Griess Reagent R2. After 10 minutes of incubation at room temperature, the color developed and the absorbance was read at 540 nm.

### Statistics.

Data were analyzed with GraphPad Prism software and were presented as mean ± SD. Statistical significance between 2 groups was assessed using 2-tailed *t* test. Differences between more than 2 groups were assessed using 1-way ANOVA and Newman-Keuls multiple-comparison test. The level of significance was set at *P* < 0.05.

### Study approval.

All procedures were performed in accordance with the guidelines approved by the IACUC at Nanjing Medical University (no. 20090053). All animal work was performed at Animal Research Center of Nanjing Medical University.

## Author contributions

ZJ, AZ, JY, and SW designed the experiments and analyzed data; JY and SW wrote the manuscript text; JY, SW, WS, WZ, and YN performed the experiments; SH and YZ contributed to the protocols and technical advices; ZJ and AZ helped draft the final manuscript; and all authors reviewed the manuscript. Order of equally contributing authors was decided based on the timeline of their contributions.

## Supplementary Material

Supplemental data

## Version 1 08/17/2021

In-Press Preview

## Version 2 09/22/2021

Electronic publication
