# Supplementary material for: Roxadustat prevents Ang II hypertension by targeting angiotensin receptors and eNOS
Source: JCI Insight. 2021 Sep 22;6(18):e133690. doi: 10.1172/jci.insight.133690 (PMC8492313; doi:10.1172/jci.insight.133690)
Supplement: Supplemental data [file jciinsight-6-133690-s105.pdf]

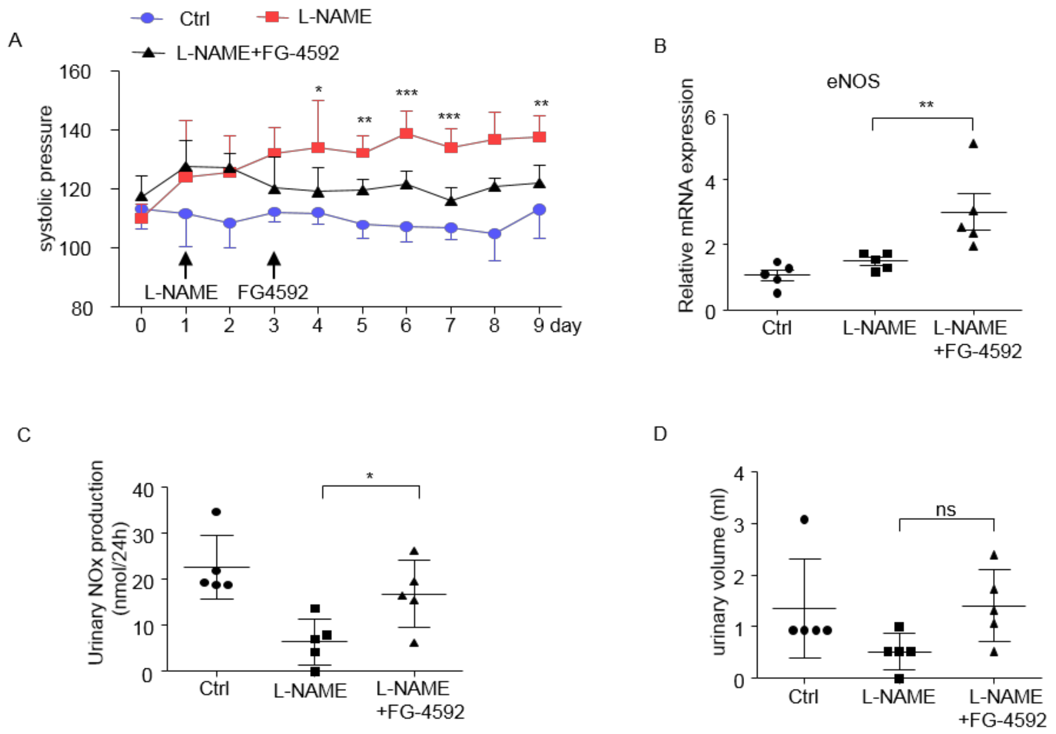

Fig. S1 L-NAME-induced hypertension is partially blunted by FG-4592 administration. (A). Systolic blood pressure measured by tail cuff for 9 days was partially blunted in the FG-4592 treatment group. L-NAME was administered in drinking water at 1.3 mg/ml. FG-4592 was given to mice after administration of L-NAME for two days. The mice treated with FG-4592 and L-NAME showed a significant decrease in systolic pressure relative to the mice treated with L-NAME alone. (B) The mRNA expression of eNOS was detected in aorta tissue by QPCR. (C) Urinary NO production. (D) urine volume. Data are presented as mean ± SD (n = 5 per group). Significance values were determined by one-way ANOVA test. Newman-Keuls multiple comparisons test was used for multiple comparison. Ctrl = DMSO in saline. $*p < 0.05$, $**p < 0.01$, $***p < 0.001$.

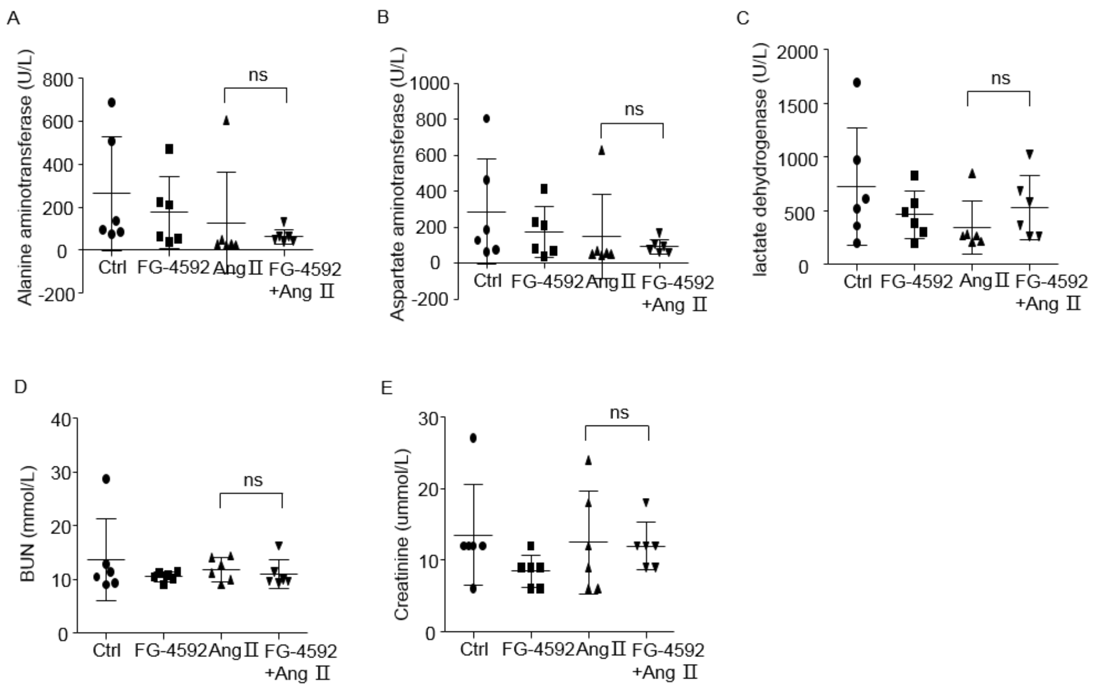

Fig. S2 Effects of FG-4592 on organ functions in mice with or without Ang II treatment. Serum ALT(A), AST(B), LDH (C), BUN (D), and Cr (E). Data are presented as mean ± SD (n = 6 per group). Significance values were determined by one-way ANOVA test. Newman-Keuls multiple comparisons test was used for multiple comparison. Ctrl = DMSO in saline.
